# Appendicostomy in Preschool Children with Anorectal Malformation: Successful Early Bowel Management with a High Frequency of Minor Complications

**DOI:** 10.1155/2013/297084

**Published:** 2013-09-23

**Authors:** Pernilla Stenström, Christina Granéli, Martin Salö, Kristine Hagelsteen, Einar Arnbjörnsson

**Affiliations:** ^1^The Department of Pediatric Surgery, Skåne University Hospital, 221 85 Lund, Sweden; ^2^Lund University, 221 00 Lund, Sweden

## Abstract

*Aim*. The aim of this study is to evaluate postoperatively bowel symptoms of antegrade colon enema through appendicostomies in preschool children with anorectal malformation (ARM). *Method*. 164 children with ARM operated on with posterior sagittal anorectal plasty were included. The malformations were classified according to Krickenbeck classification. Seventeen children in preschool age had an appendicostomy. The bowel symptoms according to the Krickenbeck follow-up were analysed pre- and postoperatively. All complications were registered. A questionnaire on the use of the appendicostomy was answered. *Results*. The median age (range) at the time of the appendicostomy was 4 (1–6) years. The observation time was 5 (0.5–14) years. The main indications for appendicostomy were incontinence and noncompliance to rectal enemas. Postoperatively there was a significant decrease in soiling and constipation (*P* < 0.001). The total complication rate was 43% with infections (29%), stenosis (12%), and retrograde leakage (0). The median time required for giving enema in the appendicostomy was 45 minutes (range: 15–120) once a day varying from 2 times/week to 3 times/day. And: complications are less frequent than in older children. *Conclusion*. Appendicostomy in preschool children with ARM is a way to achieve fecal cleanness before school start. The infection rate was high, but other complications are less frequent than in older children.

## 1. Introduction

The background to the study is the different approach to appendicostomy practiced at our institution compared with that described in the literature. In order to reach a good bowel management early on in life and with respect to the psychological aspects, we operate appendicostomies with the indication of poor compliance to rectal enemas, in young preschool children with ARM. The question is whether this can be supported by medical evidence.

Children with anorectal malformation (ARM) should be fecally clean when starting school, at the age of 5-6 years, in order to be socially accepted. The prevalence of fecal incontinence according to the Krickenbeck follow-up is reported to be 57–78% among the children with ARM [1–3]. Therefore many of these children are in need of organized bowel management, and this involves rectal enemas [4, 5]. However, compliance to rectal enemas is questionable since rectal manipulation is not well tolerated by the children who have a history of anus operations and dilatations. The psychological consequences of being subjected to rectal enemas could be difficult both for the child and the families [6, 7].

Antegrade enema through an appendicostomy was introduced in the early 1990s as an alternative to the rectal enema [8]. The main indication, besides fecal incontinence, has been to increase the child's autonomy in children who already have a good compliance to rectal enemas [9, 10]. Problems with compliance in young children with incontinence have never been described as an indication for operation. The median age in the few reports on appendicostomy in children with ARM has been 9 years [10, 11], and there are no studies about the outcome only for children at preschool ages.

The reports on the symptomatic outcome after an appendicostomy in children with ARM are few, and these describe full relief from incontinence in 96% and 72%, respectively [10, 12]. The few reports on the complications to appendicostomy in children with ARM present a relatively high complication rate of 26%–63% with strictures and fecal retrograde leakages [10, 11, 13]. The complications are reported to be more frequent among children with ARM than among other children and with more complications among younger children [13].

## 2. Aim

The primary aim of the study was to evaluate the outcome of an appendicostomy in preschool children with ARM regarding bowel related symptoms and type and frequency of complications. The secondary aim was to describe the use of and the families' satisfaction with the appendicostomy in order to prepare relevant preoperative information for the guardians.

## 3. Material and Methods

**Figure 1:**
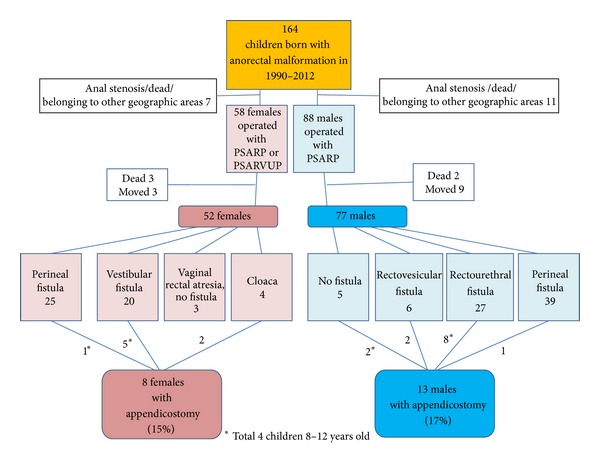
The children with anorectal malformation (ARM) operated on with posterior sagittal anorectal plasty (PSARP) and posterior sagittal anorectal vaginourethral plasty (PSARVUP) in 1990–2012 and those operated on with appendicostomies in 1998–2012. The appendicostomies among the different subtypes of ARM according to the Krickenbeck classification were unevenly distributed (*P* < 0.05, Fisher's exact test).

Since 1990, 164 children born with ARM have been admitted and initially treated at our tertiary centre for paediatric surgery with a catchment area of 2 million inhabitants. After falling off, mainly due to death or migration, the reconstructive posterior anorectal sagittal rectoplasty (PSARP) or posterior sagittal anorectal vaginal urethroplasty (PSARVUP) has been performed on 55 females and 77 males who have all been followed up at the institution (Figure 1). The type of ARM was classified according to Krickenbeck's classification, and the bowel symptoms were classified according to Krickenbeck's postoperative symptom scale [3]. The distribution of appendicostomies among the different types of ARM was statistically analysed.

The outcome and complications after the appendicostomies were registered prospectively in 2007–2012 and retrospectively in 1998–2006 through regular medical counselling and the patients' files. The bowel symptoms were statistically compared pre- and postoperatively while the complications were registered and described. 

The follow-up regarding the use of and satisfaction with the appendicostomy was registered through telephone interviews according to a questionnaire about the time required, volumes used, and parents' and children's satisfaction. The telephone interviews were carried out by doctors who were unknown to the families.

The postoperative infections were diagnosed through bacterial culturing, controlled after clinical suspicion of infection.

## 4. The Method of Operation

Appendicostomy was introduced at our centre in 1998 and has been performed with laparoscopy assistance since 2000. The procedure has been carried out or supervised by two paediatric surgeons. The laparoscopic procedure involves an open minimal laparotomy at the umbilicus with 2, 3 or 5mm 0 or 30 grade laparoscopic optic. At the site chosen for the appendicostomy, as low as possible in the right inguinal fossa, a 5 mm laparoscopy port is introduced. Through this a grasper is used to catch the appendix and pull it out through the hole of the working port. The appendix is then opened at the tip, removing 5–10 mm of the appendix. The appendiceal wall is sutured only to the skin with resorbable stiches. Until 2009, a 6- or 8-French urinary catheter was left in the stoma, and later a Chait button has been placed in the stoma. The catheter or Chait button is removed not earlier than six weeks postoperatively and in the youngest children or children with syndromes left for at least 6 months or more due to problems with accepting regular catheterising. The perioperative antibiotic prophylaxis constitutes one dose of the combination metronidazole and trimethoprim sulfa.

## 5. Statistical Analysis

As the patients were few and data could be skewed, nonparametric statistics were used. Fisher's exact Probability test, two tailed, was used for the comparisons of symptoms pre- and postoperatively and for the distribution of appendicostomy in the children with different Krickenbeck subtypes of ARM. *P* value < 0.05 was considered significant.

## 6. Ethical Considerations

The study protocol was designed to meet the legislative documentation required in the country of origin. The regional research ethics committee approved the study, registration no. 2010/49. All data are presented in such a way that it is impossible to identify any single patient.

## 7. Results

During the period from 1990 through 2012, a total of 139 children were operated on with reconstruction of the anorectal malformation at our department, and three of the children were operated on at other centres and migrated to the region. Out of these, 21 children, 1–12 years old, underwent surgery for appendicostomy (Figure 1). The distribution of appendicostomies was uneven between the different subtypes of ARM according to Krickenbeck, with the highest number of appendicostomies among the boys with rectourethral fistula (8) and the females with vestibular fistula (5). The highest rate was among the girls with cloacae (50%). In Table 1 the age of the children and the follow-up time are presented. The median age was the same in the whole group of patients 1–12 years, as in the studied group of the preschool aged children.

The preschool children's comorbidity to ARM is summarized in Table 2. None of the children were without any comorbidity, and all of them either had spinal cord malformation, neurogenic bladder or were born with a neurological syndrome.

Postoperative complications were present in 7/17 (43%) of the children. The total number of complications was 10. The type and time lapse from the appendicostomy to the complication are presented in Table 3. One patient with Mb Down had an iatrogenic subcutaneous perforation 4 months postoperatively, which had to be reoperated on. Eight out of the 17 children started with a Chait button at the time of the operation, and another two had it because of stricture. At the follow-up 8 children were still using the Chait button because of the convenience of not needing intermittent catheterising.

The pre- and postoperative symptoms according to the Krickenbeck postoperative follow-up scheme are summarized in Table 4. The results demonstrate a significant improvement of voluntary bowel movements or the ability to decide when to pass stools, as well as a significant decrease of soiling and constipation.

In three of the children the appendicostomy was allowed to close spontaneously when it is no longer needed. None had stopped using it because they did not like it, or due to any complication (Table 5).

The use of the appendicostomy is described in Table 5. As shown, the range of volumes used in the appendicostomy is wide. The volume and frequency of enema have to be tried out over a long period of time, and the results here presented are only the findings at the point of the follow-up and can change over time. Also the enemas used differed and constituted of a saline, Klyx, oil, movicol, and mixtures of these products.

## 8. Discussion

This is the first study to report the outcome of appendicostomy and its use among preschool children with ARM. The results support the approach to operate appendicostomies early, before school start. The study also shows that weak compliance to rectal enemas strengthens the indication for appendicostomy since compliance to bowel management was obtained.

Since bowel management is essential to achieve fecal cleanness both at school start and for the future bowel function [4, 14], even the psychological aspects must be respected when considering appendicostomy.

It can be pointed out that a high proportion of normal babies between the ages of 2 and three are still using diapers, passing stools 1–3 times daily. However, their use of diapers does not lead to that they are defined as incontinent and cannot be compared with the incontinent children born with ARM that are passing several stools daily and seldom are clean in between. Besides, the children here reported have a prevalence of 100% of associated malformations, especially spinal malformations, which may predict a need of appendicostomy in the near future anyway. Thus, it is justified to perform the appendicostomy operation even in one- or two-year-old babies.

The population of children with ARM belonging to the catchment area of our single institution is well controlled thanks to the national new-born register, diagnosis register, operation registers, and the national ID number. The frequency of appendicostomies was similar for the genders: 11/77 (14%) males and 6/52 (12%) females had an appendicostomy. This distribution could be questionable since the faecal incontinence among females with ARM in general is more frequent than among the males [1, 2]. One reason for the gender difference could be that the problems arising from anorectal malformation are not discussed as much with females as much as with males [15], and therefore the females may not be offered the same treatment, as appendicostomy.

The children undergoing appendicostomy are described according to Krickenbeck's classification, and this has been done once previously [10]. In comparison to that study the distribution of appendicostomies among the subtypes of ARM was similar for rectovestibular fistulas and perineal fistulas but differed for rectourethral fistulas and cloacae: 47%, 12%, respectively, in this study versus 25%, 20%, respectively, in the other study. The children with rectourethral bulbar fistulae, rectovestibular fistulae, and rectoperineal fistulae usually have an excellent functional prognosis when they receive a technically correct reconstructive procedure. However, there are exceptions to this, especially when the child has an associated malformation as spinal cord malformation or is neurologically impaired. Those children have a weaker prognosis and could therefore benefit from an appendicostomy.

In the present study, the prevalence of associated malformations with the ARM in those who underwent an appendicostomy was 100%. Spinal cord abnormalities were present in 70% and neurogenic bladder in 88%. These figures are higher than those reported among all children with ARM, where associated anomalies were present in 78% and spinal cord abnormalities in 26% [16]. Therefore it seems that anomalies in the spinal cord and urinary bladder could be predictors of a future need of appendicostomy in a new-born child with ARM.

The complication rate in this study was 43% which must be considered as high. The families with preschool children undergoing appendicostomy must be preoperatively informed about this. However, the complication rate with strictures and leakage was lower than among the previously reported older children with ARM [10, 11, 13] (Table 6). Maybe the young age could be an advantage in avoiding strictures since it seems that hypertrophic scars are less common among younger children [17]. The difference in success rate compared with what is reported before can be addressed by pointing out the young age of the children, the prospective character of the study with frequent and regular visits to the centre of pediatric surgery involved, and the difference in the duration of observations time compared with other reports. Long term results, more than 10 years, are missing.

Further, we do not use any stopper described as effective in preventing stoma stenosis in older children [18]. Instead the Chait button, earlier described for appendicostomy [19], is used for the very young children who do not accept the catheterising. The Chait button could be useful for long periods, months, or years, probably works as a stopper, and prevents stenosis. On the other hand the Chait button may be a reason for infections. A continent appendicostomy may achieve the same or better results than those obtained by us, without the use of a Chait button. However, the reason to use the Chait button is partly to avoid a stricture formation in the stoma and partly because the possible difficulties to perform catheterising in small children.

Postoperative infection was the most common complication and required antibiotic treatment in 1/3 of the patients after a median of 2-3 weeks postoperatively. Only few other studies have presented postoperative infection following appendicostomy. The reported figures are 0% in 163 patients with ARM [10] and 12–43% among children with different diagnoses, including ARM [20, 21]. The infection rate in our group of children is extremely high. This may be explained by a very close follow-up of our patients and tendency to react on minor wound problems, defining these as a treatable wound infection. On the other hand the reported figure of 0% infections in 163 patients with ARM [10] is unbelievably low in comparison to our results. At our institution the routine has been changed to an early medical control after 7–10 days postoperatively in order to start early treatment of a possible infection.

The most unexpected result was that there was no backward faecal leakage at all among the preschool children, compared to the previously reported leakage rates of 6% among ARM [10] and 21–43% among different diagnoses with a median age of 8 years [20, 21]. The reasons for the lack of retrograde leakage in young children could only be speculated on. Maybe the valve of the appendix is more continent in younger children which could explain the low incidence of leakage among the preschool children. Also the good compliance to the daily colonic wash, that was reported, could bring about low resistance in the colon, which may minimize the backward pressure and secondary leakage.

Another reason could be the operation technique. The technique used in this study is very simple without any cecal wraps or mobilization of caecum, only stitches of the appendix to the skin. In contrast to our results, the only previous study comparing complications in younger children (5.5 years) with older ones (9.1 years) used a slightly different operation technique and found that the leakage and need of manual evacuation were higher among the younger children [13]. In some studies a cecal wrap is shown to be essential to avoid a high frequency of leakage [10]; in other studies the cecal wrap is shown to be unnecessary [22].

One weakness in the study is that the symptoms were retrospectively registered in 1/3 of the patients. The information still is reliable since it was collected through contact with the parents and from the documentation of frequent notes in the files. A further weakness is that the Krickenbeck symptom scale is more suitable for the preoperative measurement and weak for the postoperative measurement, since the primary paper recommends the registration to be based on “what would the symptoms be if you/your child had no bowel management” [3]. In order to reach a balance, the postoperative questions in the study were based on the situation: “if your child only had the appendicostomy.”

Furthermore, one must consider the risk of bias when the operating surgeon is involved in a study like this one, since the result could tend to favour the operation. We are aware of this and tried to minimize the bias by delegating the follow-up to two doctors who had never met the patients and had not been the operating doctors.

The results of the questionnaires show that the time consumption for the antegrade enema is considerable and similar to earlier studies. This is a very important point in the preoperative information, especially for families who have only a little experience from rectal enemas. A good bowel management program implemented correctly takes one week. This may be true if the child and his/her guardians accept the necessary regime and stick to it. If not, a longer period of trying out the frequency and type of enema is needed. Sometimes a delayed acceptance of the duration of the enema procedure in daily life delays the compliance and the fecal cleanness. Therefore it is necessary to inform the families that it can take months until the colonic wash is well functioning because the individual amount and type of enema must be tried out, even if this has been done before with rectal enema.

The preoperative information should also include the risk of the high frequency of postoperative complications already discussed, and the fact that a Chait button is useful for a long period, sometimes years, especially in the smaller children where catheterisation could be troublesome because of their physical activity. 

The results of this study could not support the long term failure of the appendicostomies discussed in previous literature, where 40% of children have stopped using the appendicostomy after 5 years mainly because of problems [23]. Even though the follow-up time in our study was long enough: median 5 years, only 3/17 (18%) stopped using the appendicostomy. These children developed bowel control later in life after a period of being fecally clean due to the use of the appendicostomy. This does not mean that the operation was not indicated to those patients during a period of their life when they had severe incontinence problems. The reasons to why those three patients stopped using the appendicostomies were that they managed without enemas. All of them would recommend appendicostomy to others.

Earlier long term follow-ups have shown that the stenosis and reoperations will come later postoperatively, with a mean time of around 8 months [10, 24]. In our study the complications came earlier. Maybe the reasons, also to these findings, could be related to the young age.

## 9. Conclusion

The benefit of appendicostomy early on in life is that fecal cleanness could be achieved before school start and also in children who refuse rectal enemas. The complications are frequent, but stenosis and leakage less than in older children with ARM. The preoperative information about expected time and enema volumes is very important for gaining a good psychological outcome. In the end the families report a high level of satisfaction with having appendicostomy to their preschool children, and the children's autonomy could be introduced early because the appendicostomy is already in place.

## Figures and Tables

**Table 1:** Description of all the children (1–12 years) and the studied group (1–6 years) with appendicostomy.

	Number	Years median (range)
Children operated on with appendicostomy 0–12 years old	21	
Age at the appendicostomy operation		4 (1–12)
Children operated on with appendicostomy 1–6 years old	17 6 females, 11 males	
Age at the appendicostomy operation		4 (1–6)
Observation time after the operation	17	5 (0.5–14)
Age at the follow-up	17	8 (1–19)
Method used		
Laparotomy	4	
Laparoscopy	13	

**Table 2:** The comorbidity among the 17 children 1–6 years old who were operated on with appendicostomy.

Type of comorbidity	Number of children (17)
Without comorbidity	0
Spinal cord malformation, neurogenic urinary bladder, or syndromes	17
Syndrome or neurological impairment	4
Mb Down 2, autism 1, or mitochondrial disease 1	
Spinal cord malformation	10
Tethered cord 4, caudal regression 5, or skeletal anomalies 10	
Neurogenic urinary bladder	15
Urinary tract anomalies	5
Single kidney 2, VUR 5	
Cardiac malformations	5
Gynecological malformations	3
Bicorn uterus 3, cloaca 2	
Male genital anomalies	3
Hypospadia 1, undescended testicle 2	
Tracheal stenosis	1
Skeletal anomalies	3
Gastrointestinal anomaly	3
Esophageal atresia 1, duodenal atresia 1, or duodenal stenosis 1	
VACTERL	8

**Table 3:** Description of frequency and types of complications after appendicostomy in the children 1–6 years with anorectal malformation.

	Number	Time at the diagnosis in weeks median (range)	Treatment
Peroperative complications	0		
Children with postoperative complications	7 (43%)		
Incidents	10		
Types of complications			
Leakage	0		
Stricture	2 (12%)	16 (13–19)	Dilatation, Chait button
Pain	2 (12%)	14 (12–16)	Chait button
Infections	5 (29%)	3 (2–4)	Antibiotics
Iatrogenic perforation	1	18	Reoperation, Chait button

**Table 4:** Krickenbeck postoperative follow-up scheme: postoperative outcome in the 17 children with anorectal malformation operated on with appendicostomy at 1–6 years old.

Number of children *n* = 17 median aged 4 (1–6) years old		Preop. *n* 17	Postop. *n* 17	*P* value*
(1) Voluntary bowel movements, feeling of urge, capacity to verbalize, and holding the bowel movements	Yes/no	0/17	16/1	<0.001
(2) Soiling	No	2	15	<0.001
Grade 1	Occasionally (1-2/week)	0	1	
Grade 2	Every day, no social problem	2	1	
Grade 3	Constant, social problem	13	0	
(3) Constipation	No	2	14	=0.001
Grade 1	Manageable by changes in diet	2	2	
Grade 2	Requires laxative	3	1	
Grade 3	Resistant to laxative and diet	10	0	

*Fishers' exact two-tailed test.

**Table 5:** The use of the appendicostomy among the children with anorectal malformation operated on with appendicostomy at 1–6 years of age, with a median follow-up time of 5 (0.5–14) years.

Questionnaires	Answer	Median (range)	Number
Stopped using the appendicostomy (years)		5 (1.5–10)	3
Total volume enema used at each treatment (mL)		850 (200–3000)	14
Volume enema used at each treatment (mL/kg)		35 (11–80)	14
Time needed to administer the enema and finish the bowel emptying (min)		45 (15–60)	14
Frequency of the use of the appendicostomy	Once daily		10
	Three times daily		1
	Every other day		2
	Weekly		1
Appendicostomy could be recommended to others in the same situation	Yes		15
	No		2*
Use of Chait button			8

*Comments: (1) too early to evaluate and (2) were satisfied but had heard negative experiences from others.

**Table 6:** Discussion table: the types and frequency of complications with appendicostomy reported exclusively among children with anorectal malformation (ARM).

Reference	Number children	Age	No complications	Stenosis/stricture	Leakage	Surgical revision	Bowel obstruction	Infection
Kim et al. 2006 [13]	8	8.5	0%	?	?	63%	?	?
Mattix et al. 2007 [11]	32	9	?	50%	?	34%	3%	?
Rangel et al. 2011 [10]	163	9.9	74%	18%	6%	23%	0.1%	0%
Present study 2013	17	4	58%	12%	0%	6%	0	29%
